# High-Precision Measurement Method for Small Angles Based on the Defect Spot Mode of the Position-Sensitive Detector

**DOI:** 10.3390/s24227120

**Published:** 2024-11-05

**Authors:** Yusheng Zhai, Guorong Wang, Yiheng Zhao, Rongxin Wu, Lin Zhang, Zhan Su, Zhifeng Zhang, Peng Yang, Ruiliang Zhang

**Affiliations:** 1 School of Electronics and Information, Zhengzhou University of Light Industry, Zhengzhou 450000, China; 332211030780@zzuli.edu.cn (G.W.); 332011030614@zzuli.edu.cn (Y.Z.); 332311030847@zzuli.edu.cn (R.W.); 2013005@zzuli.edu.cn (P.Y.); 2017073@zzuli.edu.cn (R.Z.); 2 White Pigeon Abrasive Tools Co., Ltd., Zhengzhou 450000, China; zl8082zl@163.com; 3 Henan Academy of Sciences Institute of Applied Physics Co., Ltd., Zhengzhou 450000, China; suzhan0813@163.com

**Keywords:** photoelectric technology, angle measurement, PSD, defect spot working mode

## Abstract

The paper proposes and verifies a small-angle measurement method based on the defect spot mode of the position-sensitive detector (PSD). With the output characteristics of the PSD in the defect spot mode and the size transformation properties of a focused beam, the measurement sensitivity can be significantly improved. Calibration experiments with the piezoelectric transducer (PZT) indicate that compared with the current PSD-based autocollimation method, the proposed method can improve the sensitivity of small-angle measurement by 57 times, and the measurement sensitivity of the proposed method can be further improved by optimizing the system parameters, while the proposed method has the advantages of a simple system and high real-time performance. Therefore, the proposed method is expected to be used in high-precision motion error detection, as well as in shape and position measurement.

## 1. Introduction

High-precision angle measurement is important in many fields, such as motion error detection, shape and position measurement, etc. Due to their non-contact, real-time applicability, and high sensitivity, many optical methods have been proposed and applied. Among them, the methods based on the polarization characteristics of light [1,2], the Sagnac effect [3,4], and vortex beam transformation [5] have a larger measurement range, while the methods based on internal reflection effect [6,7], laser interference [8,9], and autocollimation [10,11,12,13,14,15,16,17,18,19,20,21] have higher sensitivity in small-angle measurement. 

The sensitivity of the internal reflection methods can reach 0.015 μrad. Such methods are based on polarized light intensity or phase detection, which is easily disturbed by factors such as the change in polarization state and stray light. The sensitivity of the laser interferometer can reach 0.03 μrad (Renishaw Corp., London, UK, XM-60). The interferometry methods represented by a laser interferometer are based on phase detection; the change in light intensity does not influence measurement results, but the influences of environmental stability need to be considered, and the interferometry system is complex and expensive. The autocollimator is the most widely used high-precision angle measuring instrument at present. Laser collimation measurement methods represented by the autocollimator are based on the detection of the spatial position of the beam. The accuracy of this kind of method is mainly determined by the spatial stability of the beam and the sensitivity of the position detection device. There are many types of position-sensitive detection devices, including the PSD, the quadrant detector (QD), and other photodiode (PD) combinations based on photocurrent detection, as well as the CCD and the CMOS based on image recognition. The sensitivity of the current PSD-based photoelectric autocollimator is approximately 0.05 μrad [10,11]. The method proposed by Gao W.’s research group, which used QD or PD photosensitive zone edges for focused spot position detection, can further improve the measurement sensitivity, reaching 0.005 μrad. The principle is to use the edge of the photosensitive area of a PD to convert the displacement of the focused spot into the energy of the partial spot entering its photosensitive area and combine it with the energy of the complete spot measured by another PD to convert it into the degree of spot defect. The way to improve the sensitivity of this method is to reduce the size of the focused spot to increase the degree of spot defect. Obviously, in order to further improve sensitivity, obtaining a smaller focused spot that breaks through the diffraction limit is a problem that needs to be solved. Meanwhile, directly utilizing the edge area of the detector for energy detection is an unconventional use of the detection device, and the performance of the edge area of the device needs to be considered [12,13]. The sensitivity of the CCD or CMOS-based digital autocollimator can even reach 0.0005 μrad (Taylor Hobson Corp., Leicester, UK, Ultra HP 142-204); however, the high sensitivity of the digital autocollimator is mainly due to the image processing algorithm [14,15,16,17,18,19]; therefore, the system is relatively complex, and the real-time responsiveness needs to be considered. 

In summary, for high-precision measurement of small angles, when comprehensively considering the measurement sensitivity, system structure, and cost, laser collimation measurement methods have more advantages. The further improvement of the sensitivity of this kind of method is mainly limited by the sensitivity of the position detection device. Although the sensitivity of the digital autocollimator is greatly improved by the image processing algorithm, the system based on image processing is relatively complex, and the real-time response is reduced.

In this study, a simple and high-precision small-angle measurement method is proposed. For the first time, the innovative PSD in defect spot working mode has been used for angle measurement, achieving ultra-high sensitivity improvement through a simple projection relationship. The proposed method is an almost entirely analog system, which has natural advantages in system complexity and real-time responsiveness compared to digital autocollimators. Meanwhile, compared with the current PSD-based autocollimation methods, the measurement sensitivity of the proposed method improved by 57 times. Compared with the method of using the detector edge to detect the degree of spot defect [12,13], the proposed method is more flexible, easier to generalize, and has greater potential for sensitivity improvement.

## 2. Detection Principle

### 2.1. The Conventional PSD-Based AutoCollimation Methods and the Defect Spot Working Mode of the PSD

The PSD can identify the position of the spot energy center in its photosensitive area. In the conventional working mode of the PSD, the complete light spot moves on the photosensitive area of the PSD; the corresponding displacement of the complete spot energy center is output linearly by the PSD [10,11].

**Figure 1:**
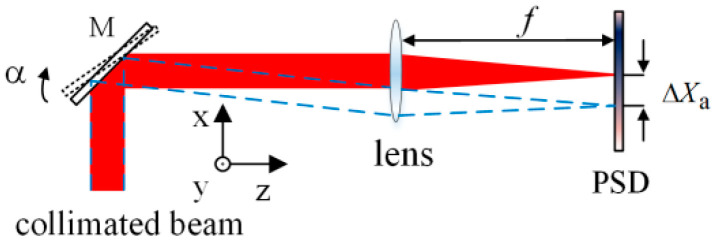
Measuring optical path of autocollimator based on conventional working mode of the PSD.

The autocollimation method based on the normal working mode of the PSD is shown in Figure 1. The collimated beam is incident on the mirror M. For any change in the angle of M, the beam deflection will be twice the rotation angle of the M, and the focused spot of the reflected light on the PSD moves accordingly. The angular change α of M can be expressed as follows:

(1) $$\alpha ={-\Delta X}_{a}/2f=k_{1}{\Delta X}_{a},$$

where f is the focal length of the lens, $\Delta X_{a}$ denotes the displacement of the light spot on the PSD caused by α, and $k_{1}$ represents the constant determined by system parameters.

**Figure 2:**
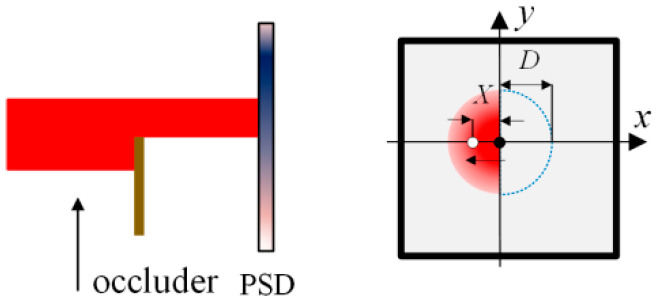
The defect spot working mode of the PSD. The black and white circles represent the spot center before and after the change of the measured beam, respectively.

The defect of the spot can also lead to the change of the spot energy center, but there are few related studies [12,22]. An innovative defect working mode [23] of the PSD is shown in Figure 2. The spot received by the PSD is a circular Gaussian spot, which is the most common shape and pattern of light spots, and its center is located at the center of the PSD. When the beam is occluded, the light spot is no longer complete; the energy center of the defect spot output from PSD changes according to the defect of light spot.

**Figure 3:**
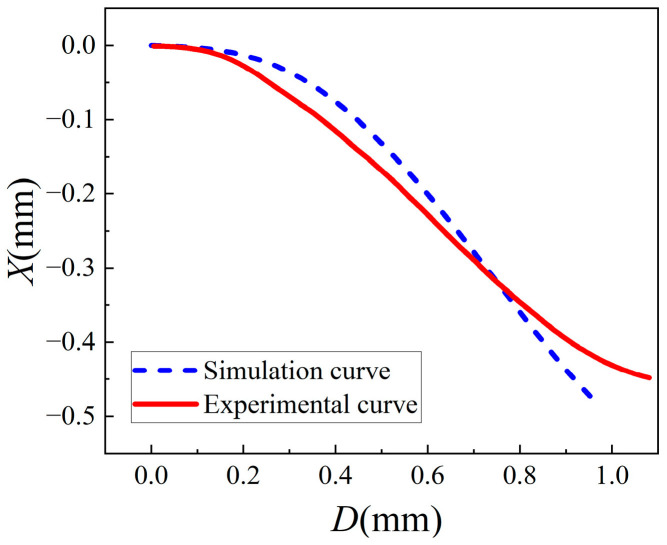
Spot energy center position output characteristics of the PSD in the defect spot mode.

As shown in Figure 2, assuming that the defect is along the *x* direction, and the width of the defect is *D*, and the spot energy center positions is *X*. The spot energy center position output characteristics of the PSD in the defect spot mode are shown in Figure 3.

It can be observed from Figure 3 that in the middle of the curve, the PSD is more sensitive, and the output is approximately linear, although for light-displacement detection, the sensitivity in the defect spot mode is lower than that in conventional working mode of the PSD shown in Figure 1. However, the defect spot mode of the PSD, combined with certain optical structures, can provide new features, which will be discussed in the next section.

### 2.2. Small-Angle Measurement Principle Based on the Defect Spot Mode of the PSD

**Figure 4:**
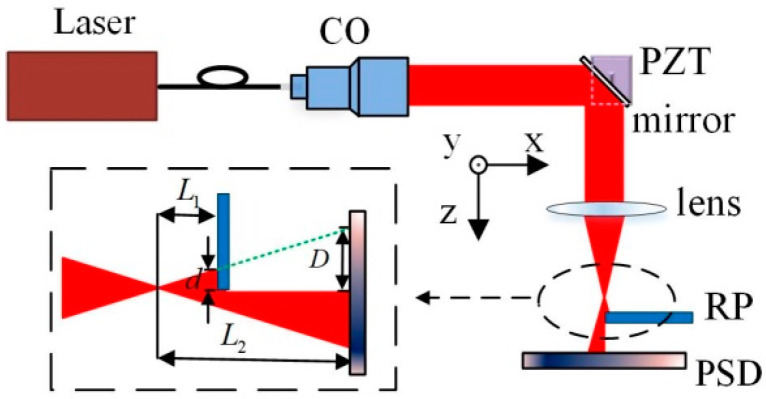
Optical path of high-precision small-angle measurement method based on defect spot mode of the PSD.

We propose a high-precision small-angle measurement method based on the defect spot mode of the PSD. The optical path structure is shown in Figure 4. A single-mode fiber-coupled semiconductor laser was used as the system light source, and the beam was collimated using a collimating objective (CO). The collimated beam was reflected by the mirror (target to be detected) mounted on the piezoelectric transducer (PZT) rotation stage and focused by a lens with focal length f. A rectangular plate (RP) that partially (about half) occluded the beam was placed near the focus point of the lens. The PSD was placed behind the focal point of the lens, and the direction in which the beam was occluded was consistent with the x-axis direction of the PSD. 

Assuming that the distance between RP and focal point of the lens is $L_{1}$, and the distance between the PSD and focal point of the lens is $L_{2}$, the relationship between the defect width *D* of the spot on the PSD and the defect width *d* of the beam occluded by RP can be expressed as follows:

(2) $$D=\left(\frac{L_{2}}{L_{1}}\right)d.$$

When the mirror rotates around the y-axis by an angle α, the focused spot moves along the x-axis. According to the optical characteristics of the lens, as shown in Figure 1, the collimated beam reflected by the mirror is focused on the intersection of the main ray and the focal plane. The overall light spot displacement of the beam on the RP plane can be expressed by the corresponding main ray displacement Δd. So, the relationship between Δd and α can be expressed as follows:

(3) $$\Delta d=2\left(f+L_{1}\right)\alpha .$$

From Equation (2), the size of the spot defect on PSD changed according to α.

(4) $$\Delta D=(L_{2}/L_{1})2(f+L_{1})\alpha$$

It is known from Figure 3 that PSD has approximately linear output characteristics within a certain range near half defect point; therefore, the change of the spot position output from PSD caused by the spot defect can be expressed as follows:

(5) $$\Delta X\left(D\right)=X^{\prime }\left(D\right)\left(\frac{L_{2}}{L_{1}}\right)2\left(f+L_{1}\right)\alpha .$$

In practice, the spot displacement output from the PSD should be expressed as follows:

(6) $$S=\Delta X\left(D\right)+\Delta X_{a},$$

where $\Delta X_{a}$ is the same as defined in Equation (1). From Figure 1, the overall light spot displacement of the beam on the PSD can be expressed by the corresponding main ray displacement $\Delta X_{\alpha }$:

(7) $$\Delta X_{a}=-2\left(f+L_{2}\right)\alpha .$$

So, Equation (6) also can be expressed as follows:

(8) $$S=2\left[X^{\prime }\left(D\right)\left(L_{2}/L_{1}\right)\left(f+L_{1}\right)-\left(f+L_{2}\right)\right]\alpha .$$

The angle change α of mirror can be expressed by the following relation:

(9) $$\alpha =S/2\left[X^{\prime }\left(D\right)\left(L_{2}/L_{1}\right)\left(f+L_{1}\right)-\left(f+L_{2}\right)\right]=k_{2}S,$$

where $k_{2}$ represents a constant determined by system parameters.

Considering the size of the PSD sensitive area, $L_{2}$ is about same as f. From Equations (5) and (7), as long as $L_{1}$ is sufficiently small, the magnification of the spot energy center displacement $\Delta X\left(D\right)$ will be very large; $\Delta X\left(D\right)$ will be much greater than $\Delta X_{a}$. Therefore, compared with the method shown in Figure 1, from Equations (1) and (9), the angle measurement sensitivity of the proposed system can be significantly improved. This amplification property is derived from the defect spot mode of the PSD, which can amplify the displacement of the spot energy center at the same magnification as the size transformation of the asymmetric defect spot.

The proposed method can be used for biaxial rotation measurements as long as the orthogonal double RP are used as the shield. In order to express this succinctly, follow-up analysis and experiments are only carried out for the single-axis rotation. 

**Figure 5:**
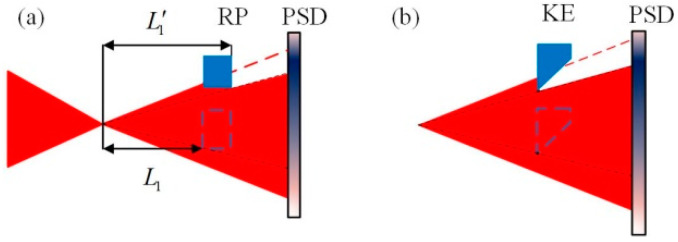
Influence of the thickness of RP. (**a**) Before adopting KE; (**b**) After adopting KE.

Because the light beam reaching the RP is not collimated, and the RP has a certain thickness, before and after the center of the light spot passes through the edge of the RP, the position of the edge of the RP that blocks the light will change. As shown in Figure 5a, the RP is placed behind the focal point, and before and after being obstructed by the RP edge at the center of the light spot, the rear and front surfaces of the RP edge respectively block the light. This implies that $L_{1}$ changes before and after the center of the spot passes through the edge of the RP. From Equation (9), it can be observed that the measurement sensitivity changes abruptly. Therefore, the edge of the RP should be selected as a knife-edge (KE), as shown in Figure 5b.

According to Equation (9), the greater the value of f, the smaller the value of$\;L_{1}$, and the higher the sensitivity of the system; at the same time, the influence of light drifts [21,24] as noise on the measurement accuracy of the system is correspondingly greater. Therefore, the parameter settings of f and $L_{1}$ need to be considered comprehensively, and the experiments described in Section 3.2 can be used as a reference.

## 3. Experiments and Analysis

**Figure 6:**
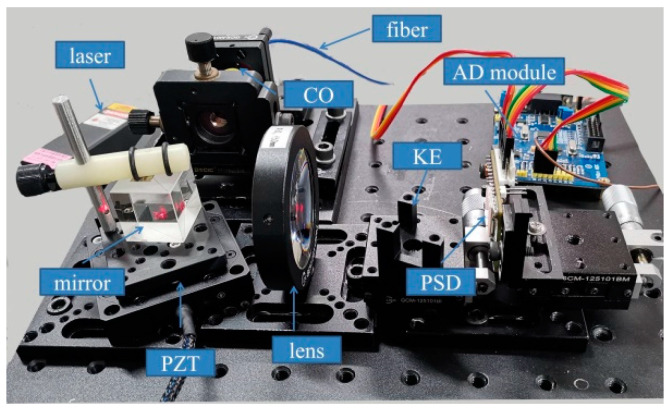
Experimental setup.

The system structure and experimental setup for high-precision small-angle measurement are shown in Figure 4 and Figure 6, respectively. The light source of the experimental system was a single-mode fiber-coupled semiconductor laser with a wavelength of 635 nm and a power of 5 mW (Xilong Optoelectronics Technology Co., Ltd., Shanghai, China, FC-635-005-SM). The laser power supply of this model is equipped with voltage and temperature stabilization modules. The diameter of the collimated beam was approximately 3.6 mm. The focal length f of the focusing lens was 50 mm, and the value of $L_{2}$ was approximately 50 mm. The beam was partially (about half) occluded by a KE, and the defect spot energy center position is detected by a PSD with a photosensitive area of 4 × 4 mm^2^ (First Senor Corp., Berlin, Germany, DL16-7-PCBA3). The AD module adopts the ADS1256 module, which has eight channels, 24 bits, and a maximum sampling frequency of 30 K, and the data are transmitted to the computer through a USB. All experimental equipment was fixed on a vibration isolation optical platform, and the experiments were conducted in a laboratory environment.

### 3.1. System Stability Experiment

**Figure 7:**
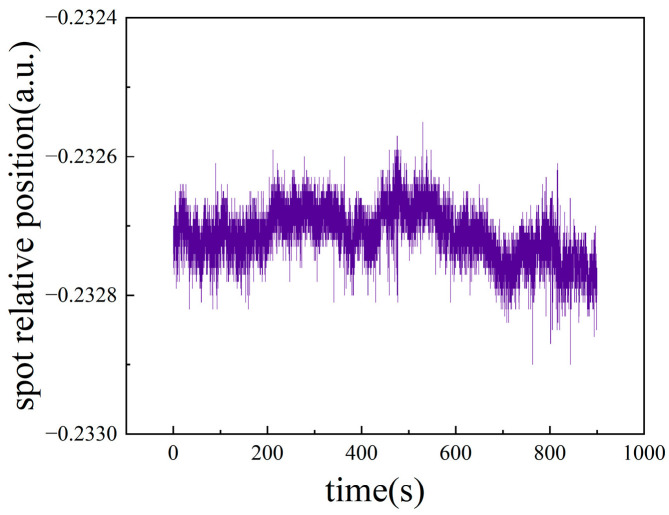
Stability experimental results.

In order to test the influence of electronic noise and environmental disturbance, the stability experiment was performed. In the experiment, the value of $L_{1}$ was approximately 3.5 mm, and the test point was chosen near the midpoint of the approximate linear working region of the PSD output characteristic curve, where the spot defect is about half and the relative position of the light spot on the PSD is 0.2327; the sampling frequency was 500 Hz, and testing time was 15 min. The experimental results after the sliding average algorithm (*n* = 10) for each sampling point are shown in Figure 7.

As shown in Figure 7, the relative position of the spot on the PSD fluctuated between −0.2329 and −0.2325. Therefore, according to the principle of significant figures in errors theory, the output values of the experimental system can be read up to 1/10,000 bit. For the selected PSD, the width of the photosensitive region is 4 mm, so a relative position change of 0.0001 corresponds to a displacement of approximately 0.2 μm. 

### 3.2. Calibration Experiments

**Figure 8:**
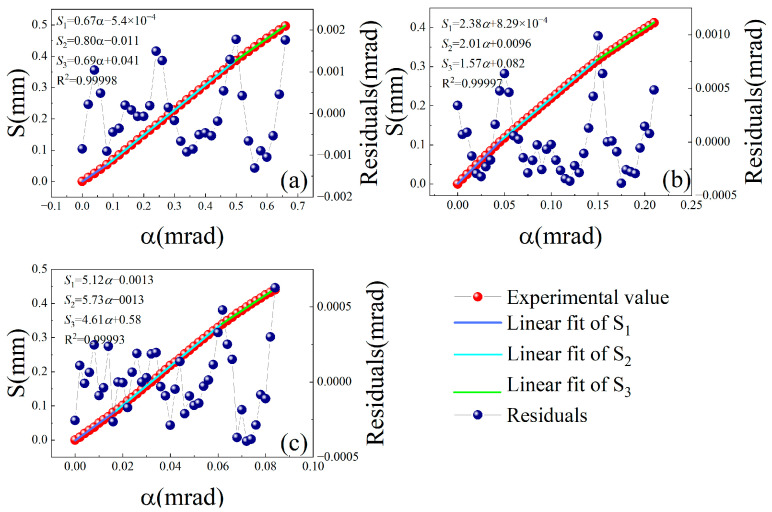
Calibration experiments of the proposed method with different values of $L_{1}$: (**a**) $L_{1}$ = 3.5 mm; (**b**) $L_{1}$ = 1.5 mm; (**c**) $L_{1}$ = 0.5 mm.

We performed several sets of small-angle measurement calibration experiments with different values of$\;L_{1}$ using the PZT rotation stage (Core Morrow Technology Co., Ltd., Harbin, China, S21.R7S) as the standard meter. Corresponding to Figure 8a–c, the values of $L_{1}$ are 3.5 mm, 1.5 mm, and 0.5 mm, respectively. The resolution, repeatability, and measuring range of the PZT rotation stage was 0.2 μrad, 0.2%, and 6 mrad, respectively. According to Equation (8) and the output characteristics of the PSD shown in Figure 3, the piecewise linear fitting method was used for the analysis and processing of the experimental data. The experimental results are shown in Figure 8.

As shown in Figure 8a, the slops of the three piecewise fitting lines $S_{1}$, $S_{2}$, and $S_{3}$ in the range of 0 mrad to 0.08 mrad, 0.08 mrad to 0.5 mrad, and 0.5 mrad to 0.66 mrad were 0.67 mm/mrad, 0.80 mm/mrad, and 0.69 mm/mrad, respectively. According to the results of stability experiment, the spot position sensitivity of the PSD was approximately 0.2 μm; therefore, the angle measurement sensitivity in the above three angular ranges were 0.29 μrad, 0.25 μrad, and 0.28 μrad, respectively. The point-to-point deviations varied from −1.3 μrad to 1.8 μrad, and the standard deviation was about 0.82 μrad. As shown in Figure 8b, the sensitivity was approximately 0.095 μrad in the range of 0.21 mrad. The point-to-point deviations varied from −0.38 μrad to 1.0 μrad, and the standard deviation was approximately 0.34 μrad. As shown in Figure 8c, the sensitivity was about 0.034 μrad in the range of 0.084 mrad. The point-to-point deviations varied from −0.39 μrad to 0.63 μrad, and the standard deviation was approximately 0.23 μrad. 

Corresponding to the three different values of $L_{1}$, the sensitivities calculated by Equation (9) are 0.26 μrad, 0.10 μrad, and 0.032 μrad, respectively; the experimental results are consistent with the theory. It can be observed from the numerical variations in the sensitivity, residuals, and standard deviation in Figure 8a–c that as $L_{1}\;$decreases, the sensitivity of the system increases, and residuals and standard deviations decrease correspondingly, but the decreasing trend of residuals and standard deviation gradually slows down, and the ratio of residuals and standard deviation to sensitivity increases gradually, which means that the relative stability of the system decreases. The residuals are mainly affected by the electronic noise and environmental disturbances, such as light drift and so on. According to the measuring principle of the proposed method, with a decrease in $L_{1}$, the influence of a factor such as light drift increases in same proportion to the sensitivity of the system, whereas the influence of other factors such as electronic noise is basically unchanged. So, the signal-to-noise ratio increases with the decrease in *L_1_*, and residuals and standard deviations correspondingly decrease. The experimental results and analysis show that the sensitivity of the proposed method can reach or even exceed 0.032 μrad, but at the same time, the standard deviation is close to seven times the sensitivity. The sensitivity determined by the results of the stability experiments, shown in Figure 7, corresponds to 1/10,000 of the relative position of the spot on the PSD. If the standard deviation is at least 10 times higher than the sensitivity, which means that the uncertainty bit of the measured value moves forward by 1 bit, the actual sensitivity of the system should correspond to 1/1000 of the relative position of the spot on the PSD. When the influence of light drift is dominant relative to the electronic noise, the further improvement of sensitivity is of limited help to the improvement of measurement accuracy. Proper compensation of light drift will be an important way to further improve the measurement accuracy of the system. Moreover, the smaller $L_{1}\;$is, the smaller the size of the light spot on KE is, so the measuring range is limited and there is a trade-off between large measuring range and high sensitivity.

We also performed a calibration experiment on the conventional autocollimation measurement method shown in Figure 1. The parameters of each device in the experimental system were the same as those in the system shown in Figure 6 above.

**Figure 9:**
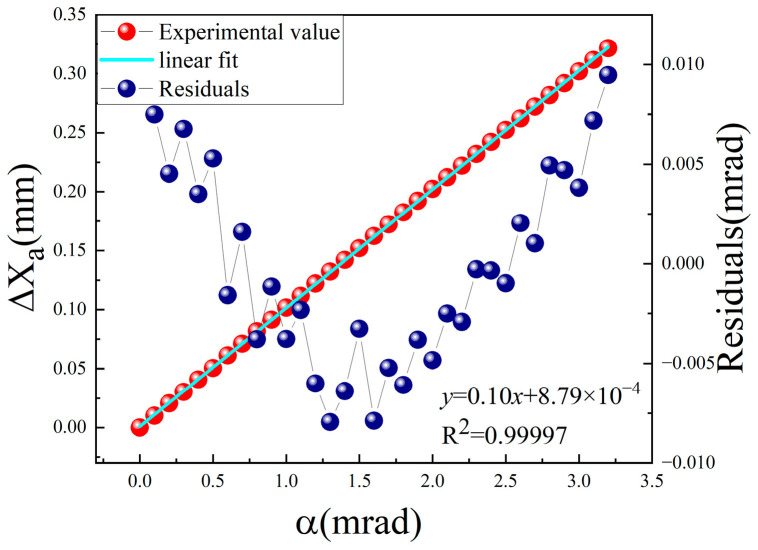
Calibration experiment of the autocollimation method based on the PSD in normal mode.

The experimental results are shown in Figure 9. In the range of 3.2 mrad, the sensitivity was approximately 2 μrad, the point-to-point deviations varied from −8.0 μrad to 9.5 μrad, and the standard deviation was approximately 5.3 μrad. 

Clearly, the method based on the conventional working mode of the PSD has a larger linear measurement range; however, the detection sensitivity of the method based on the defect spot mode of the PSD was improved by approximately 57 times, which could not be realized by conventional methods.

### 3.3. Comparison Experiments

**Figure 10:**
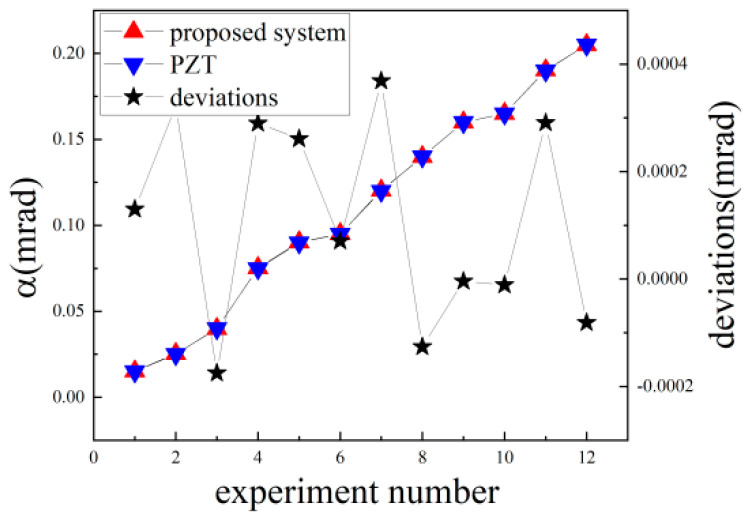
Comparison experiment results.

We conducted an angle measurement comparison experiment on the system corresponding to Figure 8b with a PZT in the rotation stage (Core Morrow Technology Co., Ltd., S21.R7S) with a resolution of 0.2 μrad as the standard meter. The PZT in the rotation stage rotates at a random angle, 12 times in total, and the corresponding measurement was performed by the proposed system. The experimental results are shown in Figure 10.

As shown in Figure 10, the deviations between the proposed system and the PZT were approximately −0.18 μrad to 0.37 μrad. Considering that the resolution of the PZT was approximately 0.2 μra, and that the standard deviation of the corresponding experiment was approximately 0.19 μrad in Figure 8b, these experimental data are quite consistent with the expectation, which confirms the feasibility and reliability of the proposed method.

### 3.4. Error Analysis

The main error sources of the proposed system include electronic noise, light drift, and the instability of the mechanical structure. Combining with Figure 4 and Equations (2)–(9), mechanical structural instability, such as the translation, rotation, or vibration of the KE, affects the numerical stability of $L_{1}\;$and *d*. Therefore, it is necessary to analyze the angular measurement errors caused by the fluctuation of $L_{1}\;$and *d*. 

According to Equations (2)–(4), the fluctuation of *d* will cause the fluctuation of *S*.

(10) $$\delta S_{d}=X^{\prime }(D)(L_{2}/L_{1})\delta$$

From Equation (9), the angular measurement error caused by the fluctuation δ*d* can be expressed as follows:

(11) $$\delta \alpha_{d}=X^{\prime }(D)(L_{2}/L_{1})\delta d/\left[2X^{\prime }\left(D\right)\left(L_{2}/L_{1}\right)\left(f+L_{1}\right)-2\left(f+L_{2}\right)\right].$$

According to Equation (9), the angular measurement error caused by the fluctuation $\delta L_{1}$ can be expressed as follows:

(12) $$\delta \alpha_{L_{1}}={SX^{\prime \left(D\right)L_{2}}f\delta L_{1}/\left[2X^{\prime \left(D\right)L_{2}}f+2\left(X^{\prime \left(D\right)L_{2}}-f-L_{2}\right)L_{1}\right]}^{2}.$$

**Figure 11:**
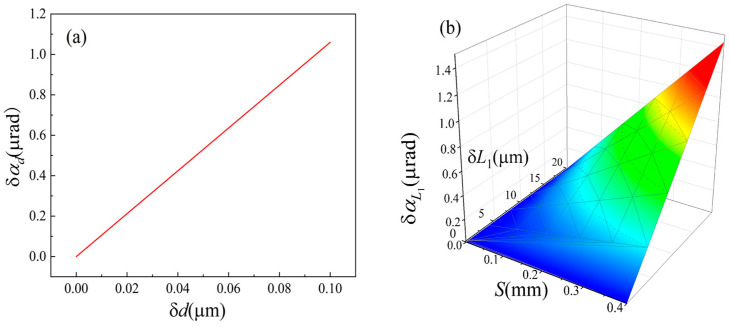
The angular measurement error caused by δ*d* and$\;\delta L_{1}$. (**a**) Error introduced by δ*d*; (**b**) Error introduced by δ*L*_1_.

The values of the structural parameters of the system corresponding to Figure 8b are substituted into the Equations (11) and (12), and the corresponding errors can be simulated by using MATLAB, as shown in Figure 11. The simulation results show that the angular measurement errors caused by fluctuations of 0.1 μm in *d* and 1 μm in$\;L_{1}\;$were 1.1 μrad and 0.038 μrad, respectively. The structural stability of KE is very important. 

Moreover, the method proposed in this paper is based on the detection of the energy center of the light spot on the PSD; the diffraction caused by the KE needs to be considered. The straight edge diffraction of the focused Gaussian beam and the point source uniform spherical wave are analyzed in references [25,26], respectively. Although the analysis processes and methods are different, the results of the two studies are similar. The closer the KE is to the focus point, the wider the diffraction fringes on the receiving plane, especially when the distance approaches 0 (the waist region); as a result, the receiving region contains only the first fringes, which leads to a symmetrical distribution of the light field, and the light field distribution based on the geometric projection relation used in proposed method is no longer applicable. So, the distance between the KE and the focus is the key to the feasibility of the proposed method.

The light field distribution of the incident light wave passing through the KE can be seen as the interference of two superimposing waves: the geometrical wave from the primary source of light and the boundary diffraction wave from the secondary source (KE) [25].

**Figure 12:**
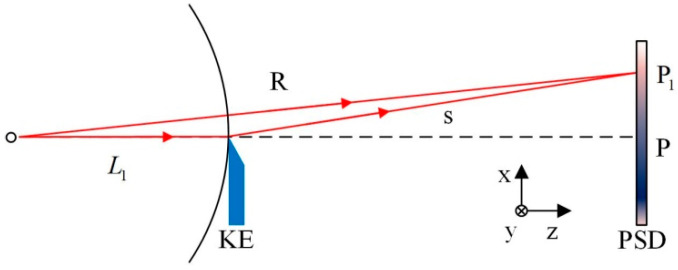
Optical structure of knife-edge diffraction.

In our case, a slightly divergent Gaussian beam is incident on KE, as shown in Figure 12. The field of Gaussian beam can be expressed as follows:

(13) $$U^{\left(g\right)}=exp[-(x^{2}+y^{2})/\omega^{2}\left(z\right)]exp\left\{-j[k(z+{(x}^{2}+y^{2})/2R)-arctan(z/F)]\right\}/\omega (z),$$

where $\omega \left(z\right)$ is the radius of the Gauss’s spot, *k* = 2π/λ denotes the wave vector, *R* is the radius of curvature of the equal phase plane of the Gaussian beam whose propagation axis intersects at the observation point, and *F* denotes the confocal parameter of the Gaussian beam. 

The boundary diffraction wave can be expressed as follows:

(14) $${U^{\left(d\right)}=\int }_{\Sigma }exp[{-l}^{2}/\omega^{2}\left(z\right)]exp\left\{-j[k(z+{s+l}^{2}/2R)-arctan(z/F)]\right\}dl/\left[\omega \left(z\right)s\right],$$

where Σ denotes the boundary of the illuminated part of the KE, dl is an infinitesimal element situated on Σ, l denotes the distance from dl to the center of the Gaussian beam profile, and s denotes the distance from dl to the observation point.

The corresponding irradiance at the observation plane can be express as follows:

(15) $${I=\left|U^{\left(g\right)}\right|}^{2}+{\left|U^{\left(d\right)}\right|}^{2}-2U^{\left(g\right)}U^{\left(d\right)}\cos\left(\varphi \right),$$

where φ denotes the phase difference between two beams.

In Equation (15), the first term represents the projection of the Gaussian beam, and the other two terms represent the diffraction term and interference term, respectively. Therefore, the light intensity distribution on the PSD can be regarded as the superposition of the direct projection spot and the disturbance spot caused by the diffraction and interference effect. In order to understand the influence of the disturbance spot on the direct projection spot, combining the system parameters with Equations (13)–(15), the intensity of direct projection light and diffraction light near the center (0.5 mm, 0) and the edge (1.5 mm, 0) of the photosensitive surface of the PSD were simulated using MATLAB. The intensity ratios of distribution light and direct projection light at the two test points, using the system parameters in Figure 8a ($L_{1}$ = 3.5 mm), were approximately 0.3% and 1.3%, respectively, and those corresponding to Figure 8c ($L_{1}$ = 0.5 mm) were approximately 0.9% and 3.5%, respectively. The simulation results show that the influence of diffraction increases with the decrease in $L_{1}$ and the increase in *x*, but the influence is weak under the given system parameters, which is consistent with the experimental results in Figure 3. Based on existing research [25,26] and the above analysis, by approximating the diffraction limit spot size to the minimum waist of the Gauss beam and combining the focused beam projection relationship with the system parameters, the minimum distance (the beam waist region) between the KE and the theoretical focus of the lens in the proposed system can be estimated to be about 0.15 mm. The experimental and simulation results show that the proposed method is feasible when the KE is not in the beam waist region.

Other factors that can change the position of the energy center of the spot can be considered an interference spot directly superimposed on the spot of the direct projection light. Because the KE and the structure of the system are invariable, the effects of the interference spot are almost invariable and can be eliminated via system calibration as system errors. The influence of random errors such as electronic noise can be partially reduced by means of the mean algorithm. 

## 4. Conclusions

This paper proposed a simple and high-precision small-angle measurement method. An innovative defect spot working mode of the PSD is used for angle measurement for the first time. Owing to the output characteristics of the PSD in defect spot mode and the size transformation properties of the focused beam, the small-angle measurement sensitivity was amplified using the same multiple as the spot size. Under the set parameters, compared with the current PSD-based autocollimation method, the sensitivity of the proposed method based on the PSD in defect mode is increased by 57 times. The main purpose of the experiments in this paper is to verify the feasibility and reliability of the proposed method, and the detection sensitivity of 0.034 μrad, given by the experiments, is not the best result of the proposed method. If the PSD with higher sensitivity [27] and the lens with larger focal length are selected and, considering the diffraction limit, the collimation system with the larger numerical aperture is selected, the sensitivity will be further improved, and it is expected to reach the level of digital collimators based on CCD and image processing algorithms, while the proposed method has the advantages of being a simple system with good real-time performance. In addition, the expansion of the measurement range is also important [13,28]. If the proposed angle measurement system is used as a “Null Detector”, and the deviation (i.e., deflection angle) of the “Null Detector” is used to modulate the position of the KE via a feedback compensation system based on a PZT, the measurement method is expected to improve through the expansion of the measurement range. This study used the most common circular Gaussian as an example. The other types of light spots have different energy distributions, and accordingly, the linear interval and slope of the PSD output characteristic curve shown in Figure 3 are different. How to obtain higher angular sensitivity gain or a larger linear measurement range via beam/edge shaping is an interesting and practical question to explore in future work. In any case, the key property derived from the defect mode of the PSD remained unchanged; that is, the magnification of the asymmetrical defect spot area on the PSD could be converted into the magnification of detection sensitivity. Due to its comprehensive advantages in sensitivity, system complexity, and real-time responsiveness, the proposed method has broad application prospects in high-precision motion error detection, as well as in shape and position measurement.

## Data Availability

Data are contained within the article.
